# Personal protection against biting insects and ticks

**DOI:** 10.1051/parasite/2011181093

**Published:** 2011-02-15

**Keywords:** vector borne diseases, personal protection, vectors, recommendations for good practice, maladies à transmission vectorielle, protection personnelle, arthropodes vecteurs, bonnes pratiques cliniques

## Abstract

Recent events with the first cases of local transmission of chikungunya and dengue fever virus in southern France by *Aedes albopictus*, adding to the nuisance and potential vectors that can be encountered when traveling in tropical or sub-tropical countries, has shown the value of a reflection on the Personal protection against vectors (PPAV). It is seen during an outbreak of vector-borne disease, or simply because of nuisance arthropods, that our fellow citizens try to protect themselves individually by using an arsenal of resources available on the market. Yet most of these means have been neither checked for effectiveness or safety tests, however, essential. Travellers, staff on mission or assignment, are looking for specific information on how to protect themselves or their families. Health workers had at their disposal so far indications that vary widely from one source to another. Therefore it seemed important to the Society of Travel Medicine (SMV) and the French Society of Parasitology (SFP) to initiate a reflection on this theme. This reflection took the form of recommendations for good practice, following the outline established by the French High Health Authority (HAS). The aim was to gather all relevant information, verified and validated and the format to be used not only by health personnel (doctors, pharmacists, nurses), but also by travel agents and individuals. This document highlights the need to take into account the risk of vector-borne diseases, some deadly, and the benefit of various methods of personal protection. The choice of methods is clearly oriented towards those whose effectiveness has been proven and potential risks assessed. The paper finally proposes two decision trees based on the transmission type (day or night) and kind of stay (short or roaming, long and steady). It concerns travellers, but also expatriates, residents and nomads.

## Abstract (fr)

L’actualité récente avec les premiers cas de transmission autochtone par *Aedes albopictus* des virus chikungunya et de la dengue dans le sud de la France continentale, s’ajoutant aux nuisances et vecteurs potentiels que l’on peut rencontrer lors de voyages ou de missions plus ou moins longues dans les régions à climat chaud, a montré l’intérêt d’une réflexion approfondie sur la Protection personnelle anti-vectorielle (PPAV). Il est observé lors d’épidémie de maladie à transmission vectorielle ou en raison simplement des nuisances liées aux arthropodes, que nos concitoyens cherchent à se protéger individuellement en ayant recours à tout un arsenal de moyens disponibles sur le marché. Or la plupart de ces moyens n’ont fait l’objet ni de contrôles d’efficacité ni de contrôles d’innocuité, pourtant indispensables. Les voyageurs, les personnels envoyés en mission ou en affectation, sont à la recherche d’informations précises sur les moyens de protection pour eux-mêmes ou leurs familles. Les personnels de santé n’avaient à leur disposition jusqu’à présent que des indications très variables d’une source à une autre. C’est pourquoi il a semblé important à la Société de Médecine des Voyages (SMV) et la Société Française de Parasitologie (SFP) d’initier une réflexion sur ce thème. Cette réflexion a pris la forme de Recommandations de bonne pratique, en suivant le schéma élaboré par la Haute Autorité de Santé (HAS). L’objectif était de rassembler toutes les informations pertinentes, vérifiées et validées, et les mettre en forme pour être utilisées non seulement par les personnels de santé (médecins, pharmaciens, infirmiers), mais aussi par les agents touristiques et les particuliers. Ce document met en exergue la nécessité de prendre en compte le risque lié aux maladies à transmission vectorielle, dont certaines sont mortelles, et le bénéfice de différentes méthodes de protection personnelle. Le choix des méthodes est clairement orienté vers celles dont l’efficacité a été prouvée et les risques éventuels évalués. Le document propose finalement deux arbres décisionnels en fonction du type de transmission (diurne ou nocturne) et du type de séjour (court ou itinérant, long et fixe). Il concerne les voyageurs, mais aussi les expatriés, les résidants et les nomades.

This recommendation has been awarded by the professional HAS. This label means that the recommendation was developed according to procedures and methodological rules recommended by the High Authority for Health (HAS).

Any dispute on the merits should be paid directly from the developer.

Cette recommandation professionnelle a reçu le label HAS.

Ce label signifie que la recommandation a été élaborée selon les procédures et régles méthodologiques préconisées par la Haute Autorité de Santé (HAS).

Toute contestation sur le fond doit être portée directement auprés du promoteur.

## Rationale

### Introduction

The chikungunya epidemics that were rife in Reunion Island in 2006 reminded the French population of the importance of fighting arthropods that serve as vectors for infectious agents. Nevertheless, the use of repellents and other protection means became gradually established in the advice to travelers for prevention of malaria and dengue, following recommendations of the “Comité des maladies du voyageur et d’importation” (CMVI), which relay the concerns of the World Health Organization (WHO). To draw a parallel with vector control^1^ (VC), this protection is named by professionals as Personal protection against vectors (PPAV) when is directed against a hematophagous arthropod (insect or tick) capable of transmitting an infectious agent.

Repelling molecules or insecticides used by the European Biocide Products Directive 98/8/EC are currently on final evaluation. Following this evaluation period for active products, final products available in the European Union will undergo a specific drug approval process.

Currently, recommendations for using PPAVs vary according to the emitting organism, both at national and international levels. This results in controversy and provides confusing information for health professionals and the public as the whole set of available products and means are available at retailers. As a result, the “Société de médecine des voyages” (SMV) and the “Société française de parasitologie” (SFP) reviewed current scientific literature on PPAVs and held a multidisciplinary discussion between experts. The consensus of the committee led to changes in the recommendations for clinical practices.

Financial support for this work was provided by the French “Direction générale de la santé”. This work has set the importance of PPAV, defines the procedure to follow at the individual level, and led to the redaction of documents targeting both health professionals and public. The recommendations are first and foremost for travelers, but also for people inhabiting areas at risk for the transmission of vector-borne diseases and nomadic populations crossing areas at risk. PPAV measures associated with vector control procedures include in-home spraying of remnant insecticides and installation of mosquito nets on windows and doors. During the preparation of this work, some observations were made that could impact regulation during the drug approval process. This collective production adds to the work coordinated by colleagues at the French “Institut de recherche pour le développement” (IRD) regarding vector control at both the adult and larva stages in France^2^.

#### General methodology

This document was written according to the method “Recommendations for clinical practice” (RCP) proposed by the French “Haute autorité de santé” (HAS)^3^.

Four bibliographic research equations were used to search the Pubmed® system and have been set up with the documentation service of the French “Agence française de sécurité sanitaire et des produits de santé”. This bibliographic research was centered on means for personal protection (*i.e.* insecticides, impregnation of clothes, mosquito nets, and repellents), the arthropods targeted, the infectious agents transmitted and the diseases they cause. The members of the workgroup used these equations with the limits inherent to their particular question. The bibliographic research also included documents from national or international institutions and/or organizations that were available on their Internet sites.

#### International organizations

World Health Organization (WHO-OMS) and their agencies, particularly the Pesticides Evaluation Scheme (Whopes) and the service for International Travel and Health.

#### National institutions

*French:* Institut de veille sanitaire (InVS), Institut national de recherche et sécurité (Inrs), Agence française de sécurité sanitaire des produits de santé (Afssaps), Agence française de sécurité sanitaire de l’environnement et du travail (Afsset), Ministère de l’écologie, de l’environnement, du développement durable et de la mer (Meeddm), Ministère des affaires étrangères (Mae), Ministère de la santé et des sports (Mss), Direction générale de l’aviation civile (Dgac-France),

*Non-French:* Centers for Disease Control and Prevention (US-CDC), Environmental Protection Agency (US-EPA), Agency for Toxic Substances and Disease Registry (US-ATSDR), Agence de réglementation de lutte antiparasitaire (ARLA – Canada), Agence de santé publique du Canada, Health protection agency (HPA – Royaume Uni), National travel health network and centre (Nathnac – Royaume Uni), Department of Health and ageing (Australie), Institut de médecine tropicale d’Anvers (Belgique).

In this document, for each addressed question, the novel recommendation and proof for each proposed measure are reported in a rating grid, adapted from Kisch^4^.

#### Evaluation of the recommendations

The proposed recommendations are rated A, B, or C according to the following criteria:

Grade A recommendations are based on results from scientific studies with a high level of proof. These studies would include comparative, randomized studies of high power without major biases, meta-analyses of comparative, randomized tests, and decision analysis based on well-performed studies (level of proof 1).

Grade B recommendations are based on a scientific presumption generated by studies with an intermediate level of proof, such as comparative, randomized tests of low power, comparative but non-randomized, wellperformed tests, and cohort studies (level of proof 2).

Grade C recommendations are based on lower-level studies, such as case-control studies (level of proof 3), or retrospective studies, series of cases and comparative studies with high-level biases (level of proof 4).

The recommendations without grade result from an agreement within the workgroup, based on their professional experience and after consultation of the reading group. They concern cases for which documentation was not available.

### What are the vectors, harms, transmitted pathogens and diseases concerned by a personal protection against vectors?

Arthropods form a vast group of extremely diverse invertebrates (Crustaceans, Arachnids, Myriapods, Insects, *etc*…), of which some species play an essential role in human pathologies. The differentiation between harmful and vector arthropods are often ambiguous: some species can successively or simultaneously belong to both categories.

The notion of harm refers to discomfort, blood spoliation, inflammation caused by stinging or biting, and allergic or dermatologic consequences related to the contact with an arthropod.

A restrictive definition states that a vector is “all hematophagous arthropod that is responsible for the active biological transmission^5^ of a pathogenic agent from one vertebrate to another.” A wider definition comprises all hematophagous arthropods that are responsible for the biological or mechanical^6^ transmission of a pathogenic agent from one vertebrate to another.

At the individual level, the tools used for protection against harmful insects and vectors are the same. However, in the risk analysis, the protection from a vector must take into account the risk related to the transmitted infectious agent. The principal vectors belong to the vast groups of insects and acari (ticks and mites).

Vector-born human and zoonotic^7^ diseases are numerous and are due to a large variety of infectious, pathogenic agents: viruses (*e.g*. chikungunya, yellow fever, dengue, *etc*…), bacteria (*e.g*. Lyme borreliosis, plague, *etc*…), protozoans (*e.g*. malaria, sleeping sickness, Chagas disease, leishmaniosis, *etc*…) or metazoans (*e.g*. loaiosis, Bancroft’s filariosis, *etc*…).

These diseases are rife mostly in tropical areas but temperate areas are not free from them. The classical triad (host, vector, infectious agent) is associated in a vectorial system that works in a particular environment that is perpetually being modified.

The following tables sum up the indispensable knowledge about vectors.

## Text of recommendations

At the individual level, prevention of vector-borne diseases needs a protection strategy against potential vectors, eventually in association with drug and/or vaccine protection. These prevention actions have been particularly studied for malaria and dengue.

### General measures for prevention

**Recommendation 1**

Due to the severity of some vector-borne diseases, analyze the risk and take into consideration measures of personal protection against vectors that would be easiest to apply. The hierarchy of these measures depends on the travel or the stay (place, season, length, modalities), and on the person (age, pregnancy, other pathology).

### Role of cutaneous repellents in PPAV

In Entomology, the commonly used definition for a repellent is, “a substance that induces an arthropod to leave”. Repellents are classified among the biocides (Directive 98/8/EC) along with insecticides, herbicides and fungicides, which represent the main families of pesticides for non-agricultural use.

After the 18th report of the WHO Expert Committee on Malaria (1986), experts recommended that repellents be used as a complement for mosquito nets and insecticide coils to reduce the human-vector contact in an individual protection strategy. In the second report from the WHO Informal Consultation (1996), the role of repellents was well defined, especially against exophagous mosquitoes and sandflies. In the 1990’s, resistance to anti-malaria drugs and insecticides led to an increased use of repellents for cutaneous use or to impregnate clothing for individual protection against vector-borne diseases. The emergence of West-Nile virus in North America led health authorities to review the strategy for protection against mosquito bites. The use of repellents was then highlighted as complementary for behavioral and environmental measures.

Thus, in the context of PPAV, a repellent is a natural or synthetic substance that has a repelling property against hematophagous arthropods. For its activity, it limits human-vector contact. With respect to the potential vector, these repellents can be classified into two categories: plant extracts and synthetic products.

The ten most ideal characteristics of a repellent are: a) long-lasting efficacy on a wide spectrum of arthropods, b) absence of skin irritation, c) lack of cutaneous absorption and toxicity, d) absence of textile fiber alterations during application on clothing, e) absence of fatty residues on the skin, f) confirmed resistance to washing and friction, g) absence of effects on common plastics, h) chemical stability, i) reasonable price for a wide use, and j) pleasant scent or lack of smell.

The use of a cutaneous repellent in PPAV has become an essential strategy to hinder arthropod biting and to fight vector-borne disease such as malaria, dengue, filariosis, *etc*… Repellents do not generally kill arthropods but modify their olfactory perception of their host.

While DEET (*N*,*N*-diethyl-*meta*-toluamide) has led the repellent market since 1946, new molecules have appeared in the recent years and improved this mode of protection because their smell is less pronounced, their texture is perceived better and they exhibit a general tolerance. Repelling molecules now in the approval process in Europe for the European Biocide Products Directive 98/8/EC are: DEET, picaridin^8^ (1- piperidin carboxylic acid, 2(2-hydroxyethyl)-methylpropylester), IR3535^9^ [3-(N-acetyl-N-butyl)aminopropionic acid ethyl ester] and PMDRBO^10^ (mixture of cis- and trans-para-menthan-3,8-diol) formely known as CitriodiolTM. Application of these products must follow some rules.

The interest in essential oils extracted from plants, as insecticides and as a potential repellent is currently expanding. Thus, a large number of extracts are studied in laboratories; however, they are complex mixtures of terpenic and aromatic derivates that vary greatly depending of the geographic area of the plant used, the manufacturer and each batch. As these extracts are very volatile, they are often used with vanillin to increase their repelling activity; this additional effect may be due to the synergy between several related molecules. The active pharmaceutical ingredients are not devoid of adverse effects, for instance citral can cause a cutaneous rash and eugenol has carcinogenic effects. Their use must be restricted to individual protection against arthropods. Two molecules were synthesized from essential oils and used for a PPAV:

1. permethrin, a synthetic pyrethrin derived from the chrysanthemum species, *Chrysanthemum* or *Tanacetum cinerariifolium*, is reserved for impregnation of clothing and mosquito nets. It exhibits both insecticidal and repellent activities;
2. Citriodiol™ (PMDRBO), derived from the eucalyptus *Corymbia citriodora* is used as skin repellent.

The benefit of using repellents against biting, hematophagous, disease-transmitting vectors override the risks of the potential severity of the transmitted diseases at all ages. The recommendation for repellent use must also be accompanied by the following measures:

1. adapt the application frequency to the principal human and vector activities present in the visited or inhabited geographic area;
2. apply on healthy, uncovered skin (useless underneath clothes);
3. do not spray directly on the face due to proven risk of eye irritation, instead spray hands and then apply onto the face, avoiding contact with peri-mucous and ocular areas;
4. rinse skin before sleep to avoid irritation by maceration in skinfolds;
5. the repellent must be applied on children by adults.

**Recommendation 2**

Only use skin repellents where the active ingredient has been evaluated as innocuous (low toxicity to animals and humans, genotoxicity, ecotoxicity) but efficient with respects to European regulation on biocidal products (Directive 98/8/EC). Follow the recommendations regarding their use. Active ingredients currently being evaluated and contained in biocidal products are: DEET, picaridin (icaridin or KBR3023), IR3535, and PMDRBO (Citriodiol™). Eventually, commercial formulas will be submitted in Europe for drug approval processes.

**Recommendation 3**

To protect from *Anopheles* during a stay in a malaria endemic area, use a commercial formulation where the active ingredient concentration is substantial enough to ensure efficient protection during at least 4 hours in field conditions, with regards to following data (Grade A).

For each product, efficient concentrations are:

- DEET: 30-50%
- IR3535: 20-35%
- Picaridin: 20-30%
- PMDRBO: 20-30%

At these concentrations, the efficiency has been shown for a longer period for *Aedes* and *Culex* species. The modalities of use must be adapted to the age and physiological conditions for each individual (child, pregnant women: see following recommendations).

**Recommendation 4**

Do not use essential oils as skin repellents because they generally have an efficacy of less than 20 minutes for principal vectors, are photo sensitive, and have high risk for allergy development (Grade B).

**Recommendation 5**

Do not apply skin repellents and sun protections at the same time. The repellent should be applied only 20 minutes after the sun protection (Grade B).

About recommendation 5, the workgroup reminds the importance of physical measures for sun protection (*i.e.* hat, protective clothing).

The workgroup proposes that:

1. The terminology used by industry to qualify finished products should be regulated. For instance:
  - A product stating a use in tropical areas should have undergone stability studies under tropical conditions and efficiency determined in the field; laboratory studies should comprise, in addition to evaluation for *Culex* and *Aedes* species, assays with one vector *Anopheles* species.
  - A product stating a use in temperate areas should have undergone efficiency studies against ticks in addition to *Culex* species.
2. Mixed products containing both repellents and sun protectents should not be available for purchase.

### Role of impregnated mosquito nets in PPAV

The use of mosquito bed nets is very old and corresponds to a simple mechanical protection that efficiently limits human-vector contact, provided that it is intact and well set. Impregnation of mosquito nets by a synthesized pyrethrinoid has demonstrated its efficacy on the decreasing the incidence of malaria at both individual and collective scales.

Impregnated mosquito nets have four recognized purposes:

1. Dissuasive effect: Less mosquitoes enter the dwelling;
2. Excito-repellent effect: mosquitoes leave more rapidly from the dwelling;
3. Inhibitory effect on feeding obtained by disturbing mosquito behavior;
4. Lethal and fast “knock down” (KD) effect.

However, with the extensive use of pyrethrinoids especially in agriculture, are resulting in an emerging:

1. Progressive mosquito selection towards pyrethrinoid tolerance, which is leading to a weak KD effect.
2. Behavioral changes of the mosquitoes.

Mosquito nets provide effective protection against many vectors with nocturnal activity. Some studies show efficiency against Chagas disease and leishmaniosis, however, few studies have looked at the efficiency for travelers.

**Recommendation 6**

- Travelers and residents should use impregnated mosquito nets to prevent malaria. The use of mosquito nets over beds should be the preferred PPAV measure for children before walking age (Grade A).
- Mosquito nets with industrial, long-lasting impregnation are preferred. However, mosquito nets conventionally or user impregnated with commercially available kits can be used as long as the the validated precautions of use are taken into consideration (Grade A).
- Perform manual impregnation methods in wellventilated spaces to avoid possible lung or eye irritation.

**Recommendation 7**

- Travelers and residents should use impregnated mosquito nets to prevent other vector-borne diseases, in particular arboviroses, Chagas disease and leishmaniosis (Grade B).
- The use of mosquito nets over beds should be the preferred PPAV measure for children before walking age.

**Recommendation 8**

Tour operators should indicate in their catalogues or leaflets the presence/absence of mosquito nets in the hosting structures. The workgroup also suggests that a follow-up card be made available for the customer (indicating the substance used and the validity date for impregnation).

### Role of impregnated clothes and fabrics in PPAV

The first synthetic repellents appeared during the Second World War and have been used for fabric impregnation. Since then, numerous technical advances have been made for repellent or insecticide molecules, fabrics and their treatment, while arthropod susceptibility to biocides have evolved. The persistence of impregnated fabric efficiency depends on numerous elements that affect the bioavailability and the persistence of the insecticide, and include: a. type of fabric, b. treatments applied, c. active ingredient formula, d. type of impregnation, e. washing method and f. UV exposure. In addition, the level of arthropod susceptibility also affects the product efficiency.

Six impregnated fabrics or supports were identified after a bibliography analysis. They are cited thereafter listed according to an increasing level of contact with skin:

1. Paper or plastic strips from Sumitomo Chemical Ltd (Osaka, Japan), which are impregnated with metofluthrin (still under study);
2. Polyethylene tarpaulin, impregnated during their production with deltamethrin;
3. Tents impregnated with insecticides, primarily pyrethrinoids;
4. Curtains impregnated with pyrethrinoids;
5. Blankets, sheets or fabrics impregnated with permethrin;
6. Impregnated clothing.

**Recommendation 9**

Wear loose and long-sleeved/legged clothes to protect against vector bites.

**Recommendation 10**

- Servicemen, foresters (Grade B), hunters, fishermen (Grade C) and also travelers should use clothing impregnated with permethrin, preferring clothes pre-impregnated during their production.
- Impregnated clothes must be combined with a skin repellent used on exposed parts of the body (Grade B) and should never replace an impregnated mosquito net while sleeping.
- Be aware of the duration of impregnation efficiency and its resistance to washing, which is low in case of manual impregnation.
- Companies that propose impregnated clothing and fabrics should indicate arthropods targeted and the duration of impregnation efficiency.

**Recommendation 11**

Residents should mount curtains impregnated with pyrethrinoids in addition to impregnated mosquito nets or when mosquito nets are not available (Grade B).

Recommendation 11 also applies to tourist resorts.

**Recommendation 12**

When no other means (mosquito nets or mosquito net-hammocks) are available, use:

- For travelers in extreme conditions or for temporary camping, polyethylene tarpaulins impregnated during their production with concentrations ≥ 360 mg deltamethrin per square meter or tents impregnated with permethrin with a concentration of 1,000 mg/m2 for an inner tent and protected by a double roof, or 2,000 mg/m2 for a simple-roofed tent (Grade B).
- For nomadic populations, a sheet, fabric pieces or blankets impregnated with permethrin at a concentration of 1,000 mg/m2 (Grade B).

Re-impregnation should be performed according to precautions of use, with validated kits that are commercially available.

### Additional measures in PPAV

Commercialized insecticides in France are synthesized pyrethrinoids or carbamates, principally designed for controlling harmful insects, and possess an immediate lethal effect with variable persistence on arthropods. Dispensed volumes during spraying with an atomizer or during passive diffusion are never standardized and always unknown by the user. A traveler, and in particular an expatriate, who wishes to buy room insecticides (in particular atomizers) is often restricted to purchase them on-site. The efficiency of local products is not guaranteed and even less is known about their innocuousness. Normal product control can vary greatly from one country to another.

Performed studies highlighted that:

1. The number of synthetic products or natural insecticides available for the general public is very important;
2. The general public does not have sufficient knowledge to use these products appropriately;
3. Instructions dedicated to the general public provide weak, wrong or undocumented suggestions regarding insects targeted, which can elicit side effects for the user and others, and/or the environment;
4. No instructions (even for so-called “natural” or “organic” insecticides) specify that “suppressing the larva nests” is a cheap (often free), efficient and ecological method (*i.e*. by eliminating standing waters around houses for mosquitoes, by vacuuming for fleas);
5. Insects targeted often have various labels on packaging, which allow the customer to understand in different manners the following terminologies: “all insects”, “harmful-flying-crawling insects”, “mosquitoes”, “special tiger mosquito”, “cockroaches”, “fleas”, “bugs”, “mites”, “special allergenic mites”, *etc*…
6. According to present knowledge, the placement of light traps cannot be specified;
7. Combustion of smoke coils releases numerous substances. Prolonged exposure to these substances are linked with some lung cancers. A recent study performed by the French AFSSET underlined the risk of unsuitable health effects due to smoke released by these coils, particularly during chronic exposure. Consequently, except during epidemics, other means are preferred especially for children, elders, asthmatics and people with respiratory disorders.

The workgroup recommends:

**Recommendation 13**

Do not “blind use” insecticides against unknown arthropods. Insecticidal control must be adapted to one or several identified arthropods.

**Recommendation 14**

Perform mechanical measures for control (destruction of larva nests, mounting of mosquito nets on windows and doors) concomitantly or before the use of chemical insecticides.

**Recommendation 15**

It is possible to use the following insecticides (though they must remain only additional measures in PPAV): atomizers for occasional use, insecticides with continuous diffusion (heating electric plugs) or in a liquid state (indoor use) (Grade B). Smoke coils must be reserved for short durations and outdoor use.

To be efficient, air-conditioning needs a good management of openings, since they constitute a limiting effect on the entrance of insects. In tropical areas, the temperatures reached (20 to 25 °C) are still compatible with vector survival and activity. Moreover, ventilation disturbs the flight of mosquitoes, which can take refuge in corners and then resume their activity once it is switched off (with or without air-conditioning). Air-conditioning and ventilation can be complemented by the use of an indoor diffusible insecticide, provided electricity supplying is continuous. Other means for personal protection are available in stores, but their efficiency has not been proved (recommendations 17 and 18).

**Recommendation 16**

Do not use air-conditioning and ventilation as the only means for PPAV. They must be complemented by airtightness of the places and by the use of insecticides to reduce the man-vector contact indoors (Grade C).

**Recommendation 17**

Do not to use anti-insect wristbands to protect from mosquitoes and ticks (Grade A).

**Recommendation 18**

Do not use an ultrasound device, vitamin B1 (Grade A), homeopathy, electric swatters, glue ribbons, papers and stickers without insecticides.

### Consideration of particular fields for PPAV

Particular fields: in the literature only studies about pregnant women, children and, to a lesser extent, lactating women can be found. There are no data about elderly or obese people. For newborns, infants and children, vectors, transmitted pathogens and diseases that concern PPAV are the same as for adults.

Pharmacological data^11^ recommend the use of repellents for children from 3 months, when the risk of severe vector-borne disease is confirmed. For safety reasons, it is proposed not to start the use of repellents before the age of 6 months; other PPAV measures should be preferred (*i.e*. impregnated mosquito nets).

For pregnant women, a study concerning the use of DEET at a 20% concentration did not provide specific maternal-fetal risks (proof level 1). Toxicological data on reproduction, clinical data and/or experience did not provide evidence that repellents were harmful during pregnancy. Pregnant women should follow the same recommendations as other adults (see recommendation 3). Length of time spent in areas subjected to transmission of vector-borne diseases should lead to a specific benefit-risk evaluation for pregnant women.

The absence of data in the literature about the use of PPAV in people with dermatitis does not allow for the identification of a secondary dermatological risk comparison to people without such lesions. Chronic, dry lesions of the skin are not a contraindication to the use of repellents.

**Recommendation 19**

- Pregnant women should use impregnated mosquito nets, prefer physical means for protection and limit exposure time to vectors, in particular at dawn and at night (Grade A).
- As toxicological data on reproduction, clinical data and/or experience did not highlight risks, pregnant women can use repellents at any stage of pregnancy when the risk of severe, vector-borne disease transmission is high. In this context, particular attention will be given to giving the lowest efficient concentration of the active ingredient. As a reminder, 4-hour efficiency against *Anopheles* in field conditions is reached with the following concentrations: DEET: 30%, picaridin: 20%, IR3535: 20%, PMDRBO: 20%.
- The use of repellents by lactating women is recommended, respecting the same precautions of use as for any adult, avoiding the breast and washing hands before lactation.

**Recommendation 20**

Children from the age of 6 months can be subjected to application of skin repellents in areas at risk for transmission of severe, vector-borne diseases.

**Table:** 

Age	Max applications per day	DEET*^1^	Picaridin	PMDRBO	IR3535^2^
6 months-walking age	1	10-30%	–	20–30%	20%
Walking age-24 months	2	10–30%	–	20–30%	20%
24 months-12 years	2	20–30%	20–30%	20–30%	20–35%
> 12 years	3	20–50%	20–30%	20–30%	20–35%

* In case of exposure to anopheles vectors of *Plasmodium*, agents of malaria, the minimum efficient concentration of DEET is 30%.

Toxicological data, clinical data and/or experience did not yet provide evidence against use in children, except in case of misuse; therefore it is recommended to use skin repellents in children from the age of 6 months in case of elevated risk from transmission of severe vector-borne diseases. In this case, the use of the minimum efficient concentration for the targeted vector must be carefully respected, as well as the maximum number of daily applications regarding the age.

1. DEET has been subjected to an assessment at the European level, usage restriction was pronounced for children before 12 years of age. However, in case of elevated risk for transmission of vector-borne disease, it can be used for a short period carefully respecting the maximum number of applications and the practical usage conditions in children.
2. Only France emitted usage restriction for IR3535 in children before the age of 30 months. This position will probably be revised according to the European assessment

Picaridin, PMDRBO and IR3535 are currently being assessed at the European level.

**Recommendation 21**

For children, respect the following precautions:

- Limit exposure time to vectors, particularly at dawn and dusk;
- Preferentially choose physical means for protection (mosquito bed nets (Grade A), or loose and impregnated protecting clothing);
- Place room insecticide dispensers away from the beds of newborns or infants;
- Prevent ingestion or projections into the eyes by keeping atomizers away from children and prevent application and manipulation of repellents and insecticides by children (Grade B);
- Do not apply repellents onto children’s hands;
- Wash body parts where repellents were applied before placing children under impregnated mosquito nets or after the end of the exposure to vectors (*i.e*. once back from a trip into the forest where there was a risk for tick bites);
- Carefully check the scalp, a common site for tick bites in children as in adults.

**Recommendation 22**

In case of extended skin lesions, use impregnated clothing (depending on the vector risk). In case of application of repellents on localized skin lesions, carefully wash repellents away, particularly DEET, as soon as the exposure to vector is over.

**Recommendation 23**

1. People with lung disorders, in particular asthma, should not use smoke coils, nor repellent or insecticide spraying and instead should use insecticide dispensers, depending on their tolerance (Grade B).
2. Additionally, they should prefer pre-impregnated fabrics (clothing and mosquito nets) and not manipulate permethrin.

**Recommendation 24**

People with contact lens should not handle their lenses after application of repellents due to the risks of irritation and eventual alteration of the contact lenses, in particular by DEET (Grade A).

### Acceptable risks associated to the use of a PPAV

Data in the literature does not identify a simple and unique indicator for the transmission capacity of the different vector-pathogenic agents. Transmission potential depends on numerous factors that entomologists group into the categories “vectorial competence” and “vectorial capacity”. Vectorial competence is the intrinsic property of a vector population to transmit a population of pathogens, as evidenced in laboratories. Vectorial capacity results from the conjugation of vectorial competence and environmental conditions: it is evaluated in the field for a chosen vector population under specific conditions. The principal of vectorpathogenic agents are well defined (see table 5), as well as vectorial competence, but the importance of the transmission of a pathogen by a vector is strongly heterogeneous due to numerous factors in relation with the host, the vectors, the pathogens and the environment.

**Decision-making diagram 1-A.** Nocturnal transmission (malaria, Japanese encephalitis, West Nile fever, leishmanioses, Chagas disease).

Short or itinerant trip			Long and fixed stay (resident, expatriate)	
Impregnated mosquito net * (++++)	OR ventilation/ air-conditioning (+) AND use of a diffusible indoor insecticide (++)	OR window and door mosquito nets (++) AND use of a diffusible indoor insecticide (++)	Impregnated mosquito net * (++++)	OR ventilation/ air-conditioning (+) AND use of a diffusible insecticide indoors (++)

Protecting clothes, ideally impregnated (++)			Intra-domiciliar spraying of remnant insecticides (+++)	

Skin repellents on exposed areas (++)			Window and door mosquito nets (++)	

Smoke coils § outdoors (+)			Impregnated clothes (++)	

			Skin repellents when outdoors (++)	

			Smoke coils § outdoors (+)	

++++: Essential; +++: Very important; ++: Important; +: Complementary; * Whenever impregnated mosquito nets are not available, use non-impregnated ones; § Out of an epidemic context of vector mosquito control, one should prefer other protection means than smoke coils, above all in children, elders, asthmatics and other respiratory disorders.

**Decision-making diagram 1-B.** Diurnal transmission (dengue, yellow fever, Chikungunya, sleeping sickness).

Short or itinerant trip		Long and fixed stay (resident, expatriate)	
Baby-bed, pushchair (…) mosquito net * for a child under walking age (++++)		Baby-bed, pushchair (…) mosquito net * for a child under walking age (++++)	

Impregnated protecting clothes ‡ (++)		Window and door mosquito nets (+++)	

Skin repellents ‡ (+++)		Electric insecticide dispenser (indoor) (++)	

Electric insecticide dispenser (indoor) (++)		Peri-domiciliar control of larva nests (++)	

Window and door mosquito nets (++)		Impregnated clothes (++)	

Air-conditioning (+)	OR impregnated mosquito net * (+) especially in epidemic situation (++)	Impregnated mosquito net *	Ventilation/air-conditioning (+)

		Skin repellents (+++)	

Smoke coils § outdoors (+)		Smoke coils § outdoors (+)	

++++: Essential; +++: Very important; ++: Important; +: Complementary; * Whenever impregnated mosquito nets are not available, use non-impregnated ones; ‡ Preferentially used for tick-borne diseases; § Out of an epidemic context of vector mosquito control, one should prefer other protection means than smoke coils, above all in children, elders, asthmatics and other respiratory disorders.

**Table I.** Principal vectors (insects and ticks).

Classes	Orders	Families	Hematophagous stages	Biology of the hematophagy	Preimaginal stages*
		*Culicidae* (mosquitoes)	Adult females	Principally crepuscular for *Aedes*;	Water
				Principally nocturnal for *Anopheles*, *Culex* and *Mansonia*	(stagnant or calm)
		
		*Simulidae* (black flies)	Adult females	Diurnal	Water (running)
		
	Diptera	*Psychodidae* (sand flies)	Adult females	Nocturnal	Land (humus, animal litters)
		
		*Tabanidae* (horse flies)	Adult females	Diurnal	Semi-aquatic
		
Insects		*Ceratopogonidae*	Adult females	Mainly crepuscular but variable among species	Land (humus)
		
		*Glossinidae* (tsetse flies)	Adult males and females	Diurnal	*In utero* except land pupa
	
	Siphonaptera (fleas)	Numerous families	Adult males and females	Several blood meals per day	Land (litters)
	
	Hemiptera Heteroptera (typical bugs)	*Reduviidae (Rhodnius, Triatoma)*	Adult males and females, and immatures	Nocturnal	Land Hematophagous
	Anoplura	*Pediculicidae* (lice)	Adult males and females, and immatures	Several blood meals per day	Land Hematophagous

		*Ixodidae* (hard ticks)	Adult males and females, and immatures	One blood meal per stage that can last several days	Land Hematophagous
		
Arachnids	Acarina	*Argasidae* (soft ticks)	Adult males and females, and immatures	Several blood meals per sage. Principally nocturnal	Land Hematophagous
		
		*Trumbiculidae (Trumbicula)*	Larvae	Lymph meal lasts several days	Land

Preimaginal stages: eggs, larvae and pupae. An image is the adult arthropod.

**Table II.** Principal traits on the comparative biology of *Anopheles*, *Aedes* and *Culex* mosquitoes.

Common points	*Anopheles*	*Aedes*	*Culex*
Hematophagy	Only adult females are hematophagous		

Number of blood meals	Each female generally takes several blood meals during its life, that can last several months		

Egg clutch	After the digestion of a blood meal, the female clutches eggs in water collections		

**Differences**	***Anopheles***	***Aedes***	***Culex***

Preferential habitat	Preferentially rural but also suburban or urban, above all in Africa	Variable according to the species, but sometimes strictly urban	

Day period of biting	Nocturnal (but some crepuscular species in South America)	Diurnal	Nocturnal

Modality of biting	A sole bite	Harasses its host until the meal is complete	Generally a sole bite

Flight type	Silent	Noisy	

Aspect of the bite	Not painful, few inflammatory signs	Sensitive with inflammatory signs of more or less extent

**Table III.** Principal methods for anti-vector protection against mosquitoes (from Carnevale, Robert *et al.* 2009)*. *In this RGP document the only methods considered and evaluated were those that reduce host-vector contact.

	Classification of methods for anti-vector protection
1) Protection technique:	. physical, biological, chemical, genetically
2) Target:	. larvae, adults
3) Effect sought by impairing:	
- host-vector contact:	. wearing of long-sleeved and long-legged clothes
	. cutaneous repellents
	. impregnated clothing (repellents-insecticides)
	. protection by domestic use of pesticides (aerosols, coils, etc…)
	. simple or impregnated mosquito nets for beds (repellents-insecticides)
- vector density:	. reduction of larva nests by modification of their environment
	. larva control with biological larvicides (larvivorous fish), biopesticides (Bacillus thuringiensis) or chemical larvicides
	. impregnated bed mosquito nets used at high scale (mass effect)
	. spatial spraying
- vector life-span:	. intra-domiciliary spraying
	. impregnated bed mosquito nets used at high scale (mass effect)
	. spatial spraying

**Table IV.** Principal vector-borne diseases with respect to geographic areas (1 = arbovirosis; 2 = bacteriosis; 3 = protozoosis; 4 = helminthiosis).

Geographic areas	Vector-borne diseases
	1. European tick-borne encephalitis; Crimean—Congo hemorrhagic fever
Northern Europe	2. Lyme borreliosis; Bartonellosis; Q fever
	3. Babesiosis

	1. West Nile fever; Toscana virus infection; Chikungunya; Dengue
Southern Europe	2. Lyme borreliosis; Boutonneuse fever (Mediterranean spotted fever); Bartonellosis; Q fever
	3. Leishmaniosis

	1. West Nile fever; Toscana virus infection
Northern Africa	2. Lyme borreliosis; Boutonneuse fever (Mediterranean spotted fever); Bartonellosis; Murine typhus; Epidemic typhus; Q fever; Pestis; Tick-borne relapsing fever
	3. Leishmaniosis

	1. Dengue; Yellow fever; Chikungunya; Rift Valley fever; Crimean—Congo hemorrhagic fever; West Nile fever
Sub-Saharan Africa	2. Tick-borne relapsing fever; African (Dutton’s) relapsing fever; Bartonellosis; Murine typhus; Epidemic typhus; Q fever; Pestis
	3. Malaria; Human African trypanosomosis (sleeping sickness); Leishmaniosis
	4. Lymphatic filariosis; Loaiosis; Onchocercosis; Serous cavity filariosis (Mansonellosis)

	1. Dengue; Chikungunya; Rift Valley fever
South-Western Indian Ocean	2. Pestis
	3. Malaria
	4. Lymphatic filariosis

	1. Dengue; Chikungunya; Crimean—Congo hemorrhagic fever; Far Eastern tick-borne encephalitis; Japanese encephalitis
Asia	2. Scrub typhus; Murine typhus; Pestis
	3. Malaria; Leishmaniosis
	4. Lymphatic filariosis

	1. Dengue; Chikungunya; Japanese encephalitis; Ross River fever
Oceania	3. Malaria
	4. Lymphatic filariosis

	1. West Nile fever; Dengue
Northern America	2. Lyme borreliosis; Rocky Mountain spotted fever; Ehrlichiosis; Pestis
	3. Babesiosis

	1. Dengue; Yellow fever; West Nile Fever
Latin America	2. Oroya fever (Carrion’s disease); Pestis; Epidemic typhus; Murine typhus
	3. Malaria; Human American trypanosomosis (Chagas disease); Leishmaniosis
	4. Serous cavity filariosis (Mansonellosis)

**Table V.** Principal human and zoonotic vector-borne infections, and their epidemiologic characteristics (non-exhaustive list). FDA = French departments in America. * documents of WHO for updated information. * Documents of WHO for updated information.

Diseases	Agents	Vectors	Hosts-reservoirs	Distribution	Mode	Incidence in transmission area	Morbidity	Lethality	Tendency

	**Virus (arbovirus)**								

Dengue	Flaviviridae Flavivirus	*Ae. aegypti, Ae. lbopictus Ae. polynesiensis*	Men, vectors	Ubiquitous (Metropolitan France, FDA, Réunion, Mayotte, Polynesia included), except cold areas	Endemo-epidemic	High	Important	Yes	Expanding

Japanese encephalitis	Flaviviridae Flavivirus	*Culex tritaeniorhynchus*	Pigs, wild birds	Indian peninsula, Far East, South-Eastern Asie, Papua	Endemo-epidemic	High	Important	Yes	Expanding except in countries that have access to vaccination

West Nile fever	Flaviviridae Flavivirus	Mosquitoes *Culex* sp.	Birds	All continents, of which Europe, Mediterranean area, Guadeloupe	Endemo-epidemic	Low	Potentially important in case of epidemics	Important in case of encephalitis	Expanding in Northern America and Mediterranean area

European tick-borne encephalitis	Flaviviridae Flavivirus	Ticks *Ixodes ricinus*	Wild mammals, vectors	Central, Eastern and Northern Europe Eastern France	Endemic	Low	Important	Mild to important in case of encephalitis	Expanding in Eastern Europe Stable in French Metropole

Far-Eastern tick-borne encephalitis	Flaviviridae Flavivirus	Ticks *I. persulcatus*	Mainly rodents	Far East and Siberia	Epidemic	Low	High	Important	Expanding

Yellow fever	Flaviviridae Flavivirus	Mosquitoes *Aedes* sp.	Monkeys, vectors	Sub-Saharan Africa, Amazon (and Guyane)	Isolated cases Epidemic	Low	Important	High	Unstable* (vaccination)

Chikungunya	Togaviridae Alphavirus	*Ae. aegypti, Ae. albopictus*	Men, monkeys, vectors	Africa, Indian Ocean (and Réunion, Mayotte), Asia, Southern Europe and France Potentially: FDA, Pacific	Epidemic	High	Important	Low	Recurrent epidemics (every 10-20 years)

Toscana virus infection	Bunyaviridae Phlebovirus	Sand flies	Men (other mammals), vectors	Mediterranean area	Endemic	Low	Mild	None	Stable but better diagnosis

Rift Valley fever	Bunyaviridae Phlebovirus	Mosquitoes *Culex, Aedes*	Ruminants, vectors	Africa, Indian Ocean (and Mayotte)	Endemo-epidemic	Currently assessed in Mayotte	Important	Low	Expanding

Crimean—Congo hemorrhagic fever	Bunyaviridae Nairovirus	Ticks *Ixodidae*, of which *Hyalomma* sp.	Wild mammals, vectors	Europe, Asia, Africa	Endemo-epidemic	Low	Important	High	Stable

	**Bacteria**								

Q fever or coxiellosis	*Coxiella burnetii*	Ticks	Mammals	Ubiquitous	Endemic	High	Important	Yes	Current epidemics in Netherlands. Stable elsewhere

Trench fever	*Bartonella quintana*	Body lice	Men	Ubiquitous	Endemic	High	Important	Yes	Expanding in homeless and highly precarious people

Oroya fever or Carrion’s disease	*Bartonella baciliformis*	Sand flies		High valleys of Andes and intertropical area of Southern America	Endemic	High	Important	Yes	Stable

Lyme disease	*Borrelia burgdorferi*	Ticks *Ixodes* sp.	Rodents, deers, birds, vectors	Northern hemisphere	Endemic	High	Important	Very low	Expanding

Tick-borne relapsing fever	*Borrelia crocidurae*	Ticks *Alectorobius sonrai*	Rodents	Western Africa	Endemic	High	Important	Low	Stable

Louse-borne relapsing fever	*Borrelia recurrentis*	Body lice	Men	Potentially ubiquitous	Epidemic	High in refugee camps	High	High	Diminishing

Epidemic typhus	*Rickettsia prowazekii*	Body lice	Men	Ubiquitous, including African mountains and Latin America	Endemo-epidemic	Variable	Important	Low	Stable

Boutonneuse fever (Mediterranean spotted fever)	*Rickettsia conorii*	Ticks *Rhipicephalus sanguineus*	Vectors, dogs, rodents	Mediterranean area including South- Eastern France	Endemic	Mild	Important	Limited	Possible expansion

Tick-borne African fever	*Rickettsia africae*	Ticks *Amblyomma* sp.	Mammals	Sub-Saharan Africa (mainly Southern)	Endemic	High	Mild	None	Possible expansion, better diagnosed

Rocky Mountain spotted fever	*R.rickettsii*	Ticks *Dermacentor* sp.	Rodents dogs	Northern America	Sporadic	Mild	Important	Yes	Expanding

Scrub typhus	*Orientia tsutsugamushi*	Mites *Trumbicula* sp.	Rodents	Far East	Sporadic	Mild	Mild	Yes	Possible expansion, better diagnosed

Pestis	*Yersinia pestis*	Fleas	Rat, Soil	Ubiquitous	Endemo-epidemic	Low, except in Madagascar and Democratic Republic of the Congo	Important	Very high if untreated (low with antibiotics)	Stable

	**Protozoans**								

Malaria	*Plasmodium* sp.	Mosquitoes *Anopheles* sp.	Men (and apes?)	Intertropical areas*	Endemo-epidemic	High	Important	High for P. falciparum	Diminishing in Guyane and Mayotte*

Babesiosis	*Babesia* sp.	Ticks	Wild mammals	Ubiquitous		Risk for splenectomized people in Europe	Important number of human cases in USA	High in Europe, low in USA	Stable

Leishmaniosis	*Leishmania* sp.	Sand flies	Mammals (including dogs)	All continents, of which Europe, Guyane and Martinique	Endemic	Low	Important	Low for cutaneous form, high for visceral form	Expanding in Guyane Influence of climate change in Europe?

Sleeping sickness 1	*Trypanosoma brucei rhodesiense*	Tsetse flies	Wild great ungulates	Eastern Africa	Endemo-epidemic foci	High	Important	Very high if untreated	Expanding

Sleeping sickness 2	*Trypanosoma brucei gambiense*	Tsetse flies	Men (and pigs?)	Western and Central Africa	Endemo-epidemic foci	High	Important	Very high if untreated	Stable

Chagas disease	*Trypanosoma cruzi*	Triatominae	Wild mammals, men	Latin America (including Guyane)	Endemic	Misestimated	Important	Important	Stable

	**Metazoans**								

Lymphatic filariosis	Filariae *Wuchereria bancrofti Brugia malayi*	Mosquitoes *Aedes, Anopheles Culex Mansonia*	Men	Africa, Indian Ocean (including Mayotte), Pacific (including French Polynesia, Wallis-et-Futuna), Asia	Endemic	Low	Maybe important and invalidating	None	Diminishing

Loaiosis	Filariae *Loa loa*	Horse flies *Chrysops*	Men	Central Africa (forest)	Endemic	Low	Important	None	Stable

Onchocercosis (river blindness)	Filariae *Onchocerca volvulus*	Black flies	Men	Western and Central Africa, Southern America	Endemic	Important	Important	None	Strongly diminishing

Serous cavity filariosis (Mansonellosis)	Filariae *Mansonella* sp.	Ceratopogonidae	Men	Western and Central Africa, Southern America	Endemic	Important	Low	None	Stable

Travelers and healthcare providers must consider whether the unsuitable effects eventually linked to PPAV are acceptable when compared to vectorial risk. For each case, the benefit-risk balance needs to be evaluated, though few direct elements are available in the literature. The workgroup has listed the relevant data about documented severe toxicity of products used in PPAV and the principal data about epidemiology, morbidity and severity of pathologies during vector-borne transmission.

According to literature:

- Acute toxicity of pyrethrinoids in normal use for PPAV is very limited and there is no evidence about long-term toxicity;
- Unsuitable, severe systemic effects of skin repellents are rare and often due to misuse. Almost all unsuitable effects of DEET, the oldest and most studied, are related to irritation of skin, mucosa, and the central nervous system.

**Recommendation 25**

The “disease” risk should be considered as superior to the “toxicity” risk of the repellents and/or insecticides when they are used following the prescribed rules.

**Recommendation 26**

During epidemics, PPAV measures should be reinforced for both residents and visitors. These measures can also lower the risk of establishing an infection in non-endemic areas for transmission of vector-borne diseases.

In malaria endemic areas, these measures must also be reinforced, particularly in the absence of prophylaxis.

In French regions particularly exposed, the workgroup suggests that the health authorities monitor exposure of residents (volumes of products consumed, analytic exposure controls).

### Long-term deleterious effects of PPAV measures

The environmental impact of the use of repellents and insecticides for PPAV has been poorly studied so far. However, ecotoxicology will be considered for all biocides in the framework of the Directive 98/8/EC, as well as the impact on health by bioaccumulation.

Environmental impacts can be defined as:

- **Non-specific** that result from the materials used for fabrication and the environmental cost. Such impacts are high for “technological” solutions, which are mostly recognized as inefficient (see R17 and R18). The products are generally manufactured overseas (environmental cost due to transportation) and made of plastics, which constitute difficult waste management.
- **Specific** impacts that are linked to the insecticidal and/or repellent molecules that are used.

Numerous publications link the health risks to the general use of pesticides mostly in agriculture but also for vector control, but the risks to health under prolonged, repeated, and regular exposures to products used for PPAV were not taken into account.

**Recommendation 27**

All products used for PPAV should:

1. Have undergone efficiency and ecotoxicology studies;
2. Not be discarded into nature after use or in case of surplus (as for the case of the re-impregnation products).

### Risks for human health in relation with harms and protection means

Besides arthropods that are vectors of pathogens, some other arthropods are pathogenic by themselves (harms due to stings or bites, myiasis, *etc*…) or serve as intermediary hosts. As a reminder, fatality due to a wasp sting is higher than due to snake bites in Metropolitan France.

The principal harms are usually caused by the following groups of arthropods:

**1) Class: Insecta**

- Order: Hymenoptera (*i.e*. bees, wasps, hornets, ants), which cause dermatological manifestations ranging from local to systemic reactions, eventually lethal.
- Order: Diptera, containing:
  - hematophagous species (*i.e*. mosquitoes, flies, horseflies, gnats), which lead to various dermatological reactions such as papular urticaria, localized regional edema, *etc*…
  - myiases agents, which are flies whose larvae develop within tissues, natural cavities or wounds. One can distinguish furuncular, migrating, wound and cavity myiases.
- Order: Anoplura (sucking lice), which cause various dermatological manifestations ranging from itching to excoriation, impetiginization, *etc*…
- Order: Psocoptera (booklice, barklice.), which causes dermatitis and respiratory allergies.
- Order: Siphonaptera, which are hematophagous fleas that provoke papular urticaria localized to lower limbs or that are diffuse, and chigoe fleas (a.k.a. jiggers) whose females burrow into the skin, leading to tumefactions in the tegument and the formation of a black furuncular nodule surrounded by a white halo.
- Order: Hemiptera:
  - hematophagous species (bedbugs, *Triatoma* sp.), whose dermatological manifestations are principally edematous papulae or localized urticaria.
  - non-hematophagous species (typical bugs), whose bites lead to a superficial burning-type vesicating effect.
- Order: Lepidoptera (butterflies); the scales of some adults or the hair of some caterpillars can provoke painful, edematous erythema, which can sometimes be bullous. An ocular topography is often observed (palpebral edema, conjunctivitis and keratitis) and some rare clinical outcomes can occur (itch and/or generalized exanthema, dyspnea, shock, disseminate intravascular coagulation).
- Order: Coleoptera, whose dermatological manifestations range from vesicular-bullous eruptions (Cantharidae) to superficial burning-typed dermatitides (Staphylinidae).

One of the main dermatological manifestations after an arthropod bite (insects or mites) is papular urticaria, whose clinical descriptions are as follows:

- Erythematous, edematous papulae from 3 to 10 mm diameter, pruriginous, sometimes accompanied by a rapidly excoriated vesicle, usually in plaques, with an irregular disposition although classically symmetrical. Duration: 2 to 10 days. Evolution to prurigo or pigmented lesion.
- Topography: usually on unprotected areas like the limbs (with some arthropods, protected areas and zones of cloth tightening). Number: a few elements to several tens.

**2) Class: Arachnida**

- Order: Araneae (spiders); the bites of some species can trigger severe dermatological manifestations (large-sized ulcers) and systemic signs.
- Order: Scorpiones (scorpions), whose bites can trigger an immediate and sometimes an intolerable burning, numbness of the region possibly accompanied by lymphangitis and adenitis. Toxin (neurotoxin) syndromes are observed in some species.
- Order: Acarina:
  - Itch mites provoke a diffuse prurit, pruriginous papules with a frequent eczema and impetigis.
  - Harvest mites (numerous species) trigger the formation of highly pruriginous papules or erythematous papulovesicles.
  - Ticks, whose bites can lead to dermatological syndromes varying from acute syndrome (hardened erythematous plaques, necrotic ulcers, bruised lesions, bullo-pustular plaques) sometimes accompanied of secondary infections, to a chronic syndrome (granu-lomatous plaque or nodule that can persist several years). Some pathogens that are transmitted by ticks can also generate pathologies with cutaneous symptoms, such as erythema migrans (Lyme disease) or eschars accompanied by regional adenopathies or lymphangitides (spotted fever-type rickettsiosis).

**3) Class: Myriapoda**

- Order: Chilopoda (centipedes); the bites of certain species can provoke a painful erythema with possible ulceration.
- Order: Diplopoda (millipedes); contact with some species can lead to vesicating lesions (superficial burns, blisters) and/or periorbital edema (conjonctivitis, keratitis).

**Recommendation 28**

The most frequent pathology observed after a journey in tropical areas is a superinfection caused by scratching lesions due to arthropod bites, in particular these of mosquitoes. Repellents have been tested alone or in association and at various concentrations, against some other harming arthropods than Culicidae: Reduviidae, Ceratopogonidae, Phlebotominae, Pediculicidae and ticks. Protection sometimes lasts more than 6 hours, but these assays must be standardized to allow a better comparison of the results and a better evaluation of their real efficiency.

To avoid known harms:

- wear protective clothing, which can constitute a physical barrier (eventually impregnated);
- sleep under a mosquito net, if possible impregnated (Grade B);
- and, if these methods are insufficient or not adapted to circumstances, use an insecticide or skin repellents, if their efficiency was demonstrated against the harming arthropod and respecting the usage recommendations (Grade A).

Currently available repellents are not efficient for a protection against hymenoptera stings (Grade B).

### Personal protection strategies according to the disease, duration of the stay and psycho-social and economical impact of the protection means

- Physical measures: according to recommendations made in France and in other countries, a consensus exists in favor of physical measures such as bed mosquito nets, protecting clothes, window and door mosquito nets.
- Children and pregnant women: documentation sources from different countries did not allow a consensus about the type and concentration of repellents that can be used in children and pregnant women.
- No consensus exists about room insecticides either. However, impregnation of mosquito nets and clothing is considered everywhere as means to reinforce the efficiency of other PPAV tools.
- The efficiency of air-conditioning is discussed (WHO, USA, Canada, and France).

To sum up, the strategies on the use and prevention measures have been poorly studied. They vary according to the vectors, the disease to prevent, the type of travelers (children, pregnant women, long-stay travelers, residents). It emerges that physical means are preferred to repellents in children and in pregnant women, and that long-stay travelers or residents have only been subjected to few specific studies.

**Two particular cases must be highlighted:**

- Malaria, for which a preventive treatment can be proposed.
- Viral diseases for which an efficient vaccine is available. In these two cases, health authorities pronounce specific recommendations.

**Recommendation 29**

For prevention of malaria, PPAV is inseparable from chemoprophylaxis recommended by health authorities (Grade A).

**Recommendation 30**

Protection against yellow fever, Japanese encephalitis and tick-borne encephalitis is based on vaccination. In case of contraindication to vaccination, absence of vaccination and if the travel cannot be delayed, use PPAV measures (Grade C). PPAV must then be the object of a written prescription.

**Recommendation 31**

Due to poor knowledge of travelers and residents, they should be informed about vector-borne diseases and about protection methods during vaccination or medical visits before departure.

Thus, to choose the optimal PPAV measures, use the two following decision-making diagrams, preferring simple messages in order to optimize the application (Decision-making diagrams 1-A et 1-B) (Grade C).

The following decision-making diagrams help orientate towards the choice of PPAV methods. During epidemics or during periods of maximal transmission, travelers, residents and expatriates should be informed about the measures in the shaded cells in Diagrams 1-A and 1-B.

Whatever the duration, itinerant tourism is classified as short stay, because the traveler will face various situations in areas with diverse epidemiological characteristics.

## Footnotes

1 Vector control : action against the vector of a pathogenic agent and not against the pathogenic agent itself. For instance: control of anopheles (the mosquito that vehiculates *Plasmodium* parasites) and not of *Plasmodium* itself (the protozoan responsible for the disease) to fight malaria

2 Fontenille D. *et al.* La lutte antivectorielle en France. IRD Editions, coll. Expertise collegiale, Marseille, 2009, 536 p. + CD-ROM.

3 ANAES. Les recommandations pour la pratique clinique. Base metho- dologique pour leur realisation en France. ANAES, Paris, 1999, 31 pages

4 ANAES. Guide d’analyse de la litterature et gradations des recom- mandations. ANAES, Paris, 2000, 60 pages.

5 Biological transmission implies the modification and/or the multiplication of the organism within the vector. Once infected during an infecting blood meal, the biological vector generally remains infected for its whole life.

6 Mechanical transmission does not imply the modification and/or the multiplication of the organism within the vector. The vector only acts as a self-mobile needle. It is generally the case during an interrupted blood meal on an infected host that is resumed rapidly on a receptive host.

7 Zoonosis is an infectious disease whose agent can be transmitted from animals to humans, formerly known as anthropozoonosis

8 Picaridin is also known as icaridine (common name) and KBR3023 (commercial name).

9 IR3535 is a commercial name accepted by WHO. This product is also called EBAAP, the acronym of its chemical formula.

10 PMDRBO (PMD Rich Botanical Oil) is purified from eucalyptus, *Corymbia citriodora;* PMD can also be produced by chemical synthesis.

